# Exploring oculomotor functions in a pilot study with healthy controls: Insights from eye-tracking and fMRI

**DOI:** 10.1371/journal.pone.0303596

**Published:** 2024-06-21

**Authors:** Ekaterina Lunkova, Sarah McCabe, Jen-Kai Chen, Rajeet Singh Saluja, Alain Ptito

**Affiliations:** 1 Department of Neurology & Neurosurgery, McGill University, Montreal, Quebec, Canada; 2 McGill University Health Centre Research Institute, Montreal, Quebec, Canada; 3 Montreal Neurological Institute, Montreal, Quebec, Canada; 4 Department of Psychology, McGill University Health Centre, Montreal, Quebec, Canada; University of Naples Federico II, ITALY

## Abstract

Eye-tracking techniques have gained widespread application in various fields including research on the visual system, neurosciences, psychology, and human-computer interaction, with emerging clinical implications. In this preliminary phase of our study, we introduce a pilot test of innovative virtual reality technology designed for tracking head and eye movements among healthy individuals. This tool was developed to assess the presence of mild traumatic brain injury (mTBI), given the frequent association of oculomotor function deficits with such injuries. Alongside eye-tracking, we also integrated fMRI due to the complementary nature of these techniques, offering insights into both neural activation patterns and behavioural responses, thereby providing a comprehensive understanding of oculomotor function. We used fMRI with tasks evaluating oculomotor functions: Smooth Pursuit (SP), Saccades, Anti-Saccades, and Optokinetic Nystagmus (OKN). Prior to the scanning, the testing with a system of VR goggles with integrated eye and head tracking was used where subjects performed the same tasks as those used in fMRI. 31 healthy adult controls (HCs) were tested with the purpose of identifying brain regions associated with these tasks and collecting preliminary norms for later comparison with concussed subjects. HCs’ fMRI results showed following peak activation regions: SP–cuneus, superior parietal lobule, paracentral lobule, inferior parietal lobule (IPL), cerebellartonsil (CT); Saccades–middle frontal gyrus (MFG), postcentral gyrus, medial frontal gyrus; Anti-saccades—precuneus, IPL, MFG; OKN—middle temporal gyrus, ACC, postcentral gyrus, MFG, CT. These results demonstrated brain regions associated with the performance on oculomotor tasks in healthy controls and most of the highlighted areas are corresponding with those affected in concussion. This suggests that the involvement of brain areas susceptible to mTBI in implementing oculomotor evaluation, taken together with commonly reported oculomotor difficulties post-concussion, may lead to finding objective biomarkers using eye-tracking tasks.

## Introduction

Eye-tracking techniques have found extensive application across diverse fields such as research on the visual system, neurosciences, psychology, and human-computer interaction. Their versatility has led to emerging clinical implications, particularly in the assessment of oculomotor deficits. In this preliminary phase of our study, we introduce a novel virtual reality technology tailored for tracking head and eye movements among healthy individuals. This innovative tool was specifically developed to assist with assessing the presence of mild traumatic brain injury (mTBI), acknowledging the frequent occurrence of oculomotor function deficits following such injuries [1,2].

Concussed individuals often complain of oculomotor symptoms, including blurred vision, convergence insufficiency, difficulty reading, diplopia, headaches, difficulty tracking a moving target, general asthenopia (eye strain), dizziness, nausea, and problems scanning visual information [3,4] which can be explained by the fact that concussion may disrupt the underlying neurophysiology of oculomotor functions [5]. In particular, mTBI may be a leading cause of clinically impaired smooth pursuit and saccadic eye movements [6].

While eye-tracking provides precise measurements of eye movements and gaze behaviour, fMRI allows for the simultaneous examination of neural activation patterns in response to specific tasks. By combining these modalities, we can obtain a comprehensive understanding of both the neural underpinnings and behavioural manifestations of oculomotor function.

During saccadic eye movements, fMRI activation is observed in frontal eye fields (FEF), supplementary eye fields (SEF), parietal eye fields (PEF) and vermis of the cerebellum, as well as in subcortical areas, such as the substantia nigra pars reticulata (SNpr), caudate nuclei (CN) and superior colliculi (SC) [7–10]. When performing anti-saccades, additional higher cognitive processes are required; they involve changes in activity levels within the basic saccade circuitry as well as recruitment of additional areas, such as the prefrontal cortex [11,12].

Studies using fMRI during Smooth Pursuit demonstrated involvement of such functional areas as SEF, FEF, MT, the vermis of the cerebellum, and vestibular nuclei [10]. Smooth Pursuit eye movements are considered to be one of the components in Optokinetic Nystagmus (OKN). In OKN, the circuit for the slow phase of the eye movement is believed to overlap with smooth pursuit, while the fast phase relies on the nucleus of the optic tract (pretectum) [13].

Eye-tracking systems offer a promising avenue for rapid, objective, and non-invasive concussion diagnosis by detecting subtle oculomotor abnormalities. Recent studies have demonstrated correlations between eye-tracking metrics and concussion symptoms, highlighting their potential as sensitive screening tools [14]. Yet, the comprehensive exploration of oculomotor metrics and their relationship to brain injury location remains limited.

In this study, we focus on healthy controls to elucidate the neural basis of oculomotor function using fMRI during tasks evaluating smooth pursuit, saccades, and optokinetic nystagmus (OKN). By examining brain activation patterns in healthy individuals, we aim to establish a baseline for future comparisons with concussed subjects [15]. This approach will provide valuable insights into the underlying mechanisms of oculomotor function and its vulnerability to mTBI.

In this study, we aimed to:

1. examine blood oxygen level dependent (BOLD) fMRI alterations corresponding to performances in oculomotor function after mTBI (evaluating saccades, anti-saccades, smooth pursuit and OKN) in healthy control subjects,
2. investigate the possibility of using virtual reality (VR) goggles with built-in eye-tracker system and a specially developed software system to automatically assess oculomotor functions in complementary tracking tasks.

Our study aims to explore whether eye-tracking metrics can serve as a potential screening tool for concussed patients in acute stage. To validate the use of eye-tracking metrics in concussion diagnosis, we need to establish the neural correlates of oculomotor tasks in healthy controls, which is why we are utilizing fMRI in conjunction with eye-tracking.

The future purpose is validating this test design as a diagnostic tool in concussed patients in the acute stage. Here, we present the results of a pilot study with healthy controls to identify brain regions associated with these tasks and to collect norms for future comparison with concussed subjects.

## Methods

### 2.1. Ethics statement

We obtained approval for this study from McGill University Institutional Review Board. Written informed consent was obtained from all participants involved in the study. The consent form outlined the purpose of the research, the procedures involved, and the potential risks and benefits. Participants were informed of their right to withdraw from the study at any time without consequences.

### 2.2. Participants

A group of 31 adult healthy control subjects (15 males and 16 females) aged 18 to 55 (mean age = 30.6, SD = 9.4) were included in the study. All were screened to be without a history of neurodevelopmental or neurological disorders, or head injuries, ADHD, and/or presence of any significant abnormalities seen on structural MRI scans (assessed by a clinician).

### 2.3. Oculomotor functions assessment using the VR-goggles eye-tracking system

Oculomotor evaluation was conducted prior to MRI scanning using virtual reality (VR) goggles with NeuroFlex® software equipped with binocular recordings in 3D (horizontal, vertical, and pupil size) and head recordings in 6D (3D angular and 3D linear accelerations). These were recorded concurrently for eye and head angles at a 120 Hz sampling rate. A high-speed laptop computer generated the goggle visual displays and recorded the ocular and head data synchronously. Eye and head movements were evaluated in response to visual and vestibular stimuli, or lack thereof (e.g., to evaluate spontaneous nystagmus) and to detect deviations from the ‘normal’ eye and head responses of the healthy subjects. Table 1 summarizes the methods and metrics evaluated. The full evaluation consisted of a battery of tests that takes less than 10 minutes to administer, including three head-free conditions (Smooth Pursuit (head-free), Active Visual Vestibulo-Ocular Reflex (VOR, Horizontal), Active Visual VOR (Vertical)) and five head-fixed conditions (Smooth Pursuit (head-fixed), Saccades, Anti-saccades, Optokinetic Nystagmus (OKN), Spontaneous Nystagmus).

**Table 1:** Eye-Head coordination tests and measured variables with units.

	System of interest (protocol)	Measured aspect of Metrics
1	Saccades (flashed targets, self-paced)	Delay (ms) Accuracy (degrees)
		Generation rate (S/sec) Main sequence (peak velocity vs. duration)
2	Anti-saccades	Accuracy (degrees)
		Latency (ms)
3	Active head-fixed or head-free passive VOR active VOR, pursuit, OKN	Mean vergence over the whole test period (sac/min)
		Vergence for each phase of movement (saccade and fixation; degrees)
4	Nystagmus during active gaze shifts head-fixed or head-free Spontaneous nystagmus in the dark Vestibulo-Ocular Reflex Optokinetic Nystagmus	Asymmetry of peak response, phase lag (%)
		Full response characterization in both phases with numeric parameters
		Generation frequency
		Tracking error, gaze stabilization (degrees)
5	Head free gaze shifts	Eye vs head contributions
6	2D Target tracking head-fixed or head-free smooth pursuit and corrective saccades	Accuracy in different initial positions (degrees)
		Corrective saccade rate (S/sec)
		Response symmetry
7	Pupil size	Diameter (mm)

After subjects underwent evaluation using VR goggles, four tasks selected: Smooth Pursuit, Saccades, Anti-Saccades and OKN–were repeated during fMRI sessions to measure brain activation associated with performance of each task. The 4 tasks during fMRI were identical to the tasks during the evaluation using VR goggles and were presented to the subjects via projector in the MRI room.

### 2.4. Image acquisition

All scanning was performed on a Siemens 3 Tesla MRI system equipped with a 64-channel head coil at the Montreal Neurological Institute (MNI) BIC MRI platform. First, T_1_-weighted images were acquired for anatomical reference (3D MP-RAGE, TR = 2300ms, TE = 2.98ms, 176 slices, slice thickness = 1mm, FOV = 256mm, image matrix = 256 x 256, flip angle = 9 degrees, interleaved excitation) for fMRI data. fMRI data was acquired using BOLD activation studies with T2* weighted GE-EPI (TR = 3000ms, TE = 30ms, 38 slices, slice thickness = 4mm, FOV = 256mm, image matrix = 128x128, interleaved excitation).

### 2.5. Oculomotor tasks used in fMRI

We used task-based fMRI with 4 tasks evaluating oculomotor functions: (1) **Smooth Pursuit**: subjects were asked to follow a moving target (dot) with their eyes; (2) **Saccades:** subjects were warned that the dot will jump around the screen, and that they had to follow it with their eyes only; (3) **Anti-saccades**: subjects had to look at the dot at the center of the screen–when a red X appeared, they had to avoid looking at the red X and instead orient their eyes in the opposite field of view in the same location–then follow the dot back to the center; (4) OKN: subjects were asked to pick a dot and follow it until it left their field of view, and to continue in the same manner with each subsequent dot; and, (5) **Baseline condition**: a) Prior to each task, the baseline condition was presented to the subjects (for conditions (1), (2) and (3) it was a fixed dot in the center of the screen for a duration of 12 seconds; for condition (4), it was a fixed field of dots for a duration of 15 seconds). Each of the conditions lasted 30 seconds, while subjects were head-fixed and asked to complete the tasks moving their eyes only. Two identical functional scanning sessions were conducted sequentially. Each scanning session lasted 6 minutes and consisted of two runs of the set of the 4 tasks. The subjects had extensive training prior to the scanning to ensure familiarity with the tasks. These tasks were chosen as participants’ head is fixed during MRI scanning and these tasks don’t require head movement as other head-free conditions in the screening battery (Head-Free Smooth Pursuit, VOR Vertical and Horizontal). Only one out of five head-fixed tasks wasn’t replicated during fMRI–Spontaneous Nystagmus, due to recent addition of this task to the screening and lack of normative data for this task.

### 2.6. Behavioral analysis (Oculomotor & Gaze assessment)

The VR-goggles in the eye-tracking system automatically gather and process the data through the NeuroFlex® software system. The results for each subject included all metrics demonstrated in Table 1, and the deviations of the results (if any) were indicated in the reports. Mean values, standard deviation (SD), and normative range of the results in the group of healthy controls were calculated. The normative range was counted as Mean+/-2SD.

### 2.7. fMRI processing

All MRI images were preprocessed and analyzed using SPM12 (https://www.fil.ion.ucl.ac.uk/spm/software/spm12/). During the preprocessing stage, all the functional images were realigned and unwrapped; slice-time corrected; co-registered to a T1-weighted reference image; structurally and functionally normalized and segmented into gray matter, white matter, and CSF tissue; and smoothed using a 6 mm Gaussian kernel. The preprocessed images for each subject then underwent first-level analysis, where the model was specified and estimated for the two runs of the tasks. The model was specified using the conditions and onset times for each task. To determine an alteration of the level of BOLD signal specific for each task, the contrasts were identified between the task condition (separate for each of the four tasks) and the baseline condition. Afterwards, a second level analysis was conducted to identify BOLD signal changes during each task respectively for the healthy controls. Exploratory brain analysis resulted in whole brain activation maps. All the comparisons were subjected to Family-wise error rate (FWER) correction at p < 0.05, and only changes filtered out by the correction were considered significant. The changes were considered significant at p < 0.05. Regions of interest (ROIs) were then identified for each task using the MarsBaR toolbox [16]. Each ROIs was created in a sphere shape with a radius of 5mm. The percent BOLD signal change was calculated for the acquired ROIs.

### 2.8. Comparison between oculomotor metrics and BOLD signal alterations

Oculomotor and BOLD signal analyses and descriptive statistics were performed using the IBM Statistical Package for the Social Sciences (SPSS) version 28.0.1.1 for Mac OS. A multiple regression analysis was used to determine the existence of a relationship (if any) between level of BOLD signal change in each ROI and each oculomotor metric of the four tasks used in fMRI. For each task, multiple comparisons were made, with the BOLD signal change for each ROI of a given task as the independent variables and a metric from a given task as the dependent variable. The same analysis was repeated for each oculomotor metric.

## Results

### fMRI results

**Fig 1:**
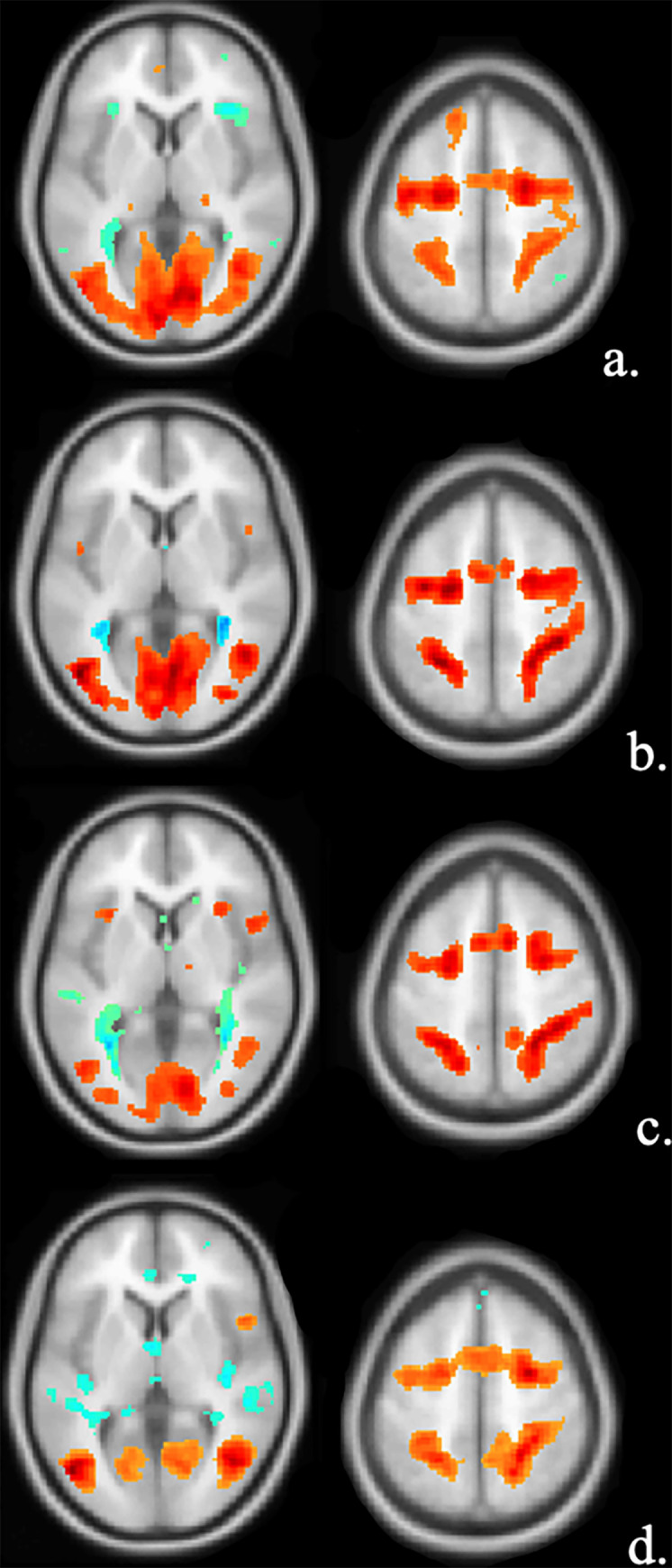
Activation during the oculomotor tasks.

Analysis of the fMRI data from the group of healthy controls detected various task-related activation foci (Fig 1). The anatomical location of the activation peaks was identified by superimposing activation maps onto the high-resolution anatomical T1-weighted image in MNI space (Table 2). All the activation peaks showed elevated BOLD signals. During the Smooth Pursuit task, there was an increase in activation in the cuneus, superior parietal lobule, paracentral lobule, cerebellar tonsil, and inferior parietal lobule. In the Saccades task, increased BOLD signals were observed in the middle frontal gyrus, postcentral gyrus, and medial frontal gyrus. In the anti-saccades task, there was an increase in BOLD signal in the precuneus, inferior parietal lobule, and middle frontal gyrus. Finally, in the Optokinetic Nystagmus task, increased activation was seen in the middle temporal gyrus, precuneus, cerebellar tonsil, anterior cingulate cortex, postcentral gyrus, and middle frontal gyrus. These fMRI results were associated with normal performances on the oculomotor tasks as indicated by the data collected with the eye-tracker goggles prior to MRI scanning.

**Table 2:** MNI coordinates and T- and p-values for each activation peak based on the whole brain analysis results and change in BOLD% for each activation peak according to ROIs analysis.

Task	Peaks	Hemisphere	x	y	z	T value	p value	Average BOLD signal % increase
Smooth Pursuit	Cuneus	Left	-10	-98	14	10.91	0	
	BA7 (Superior parietal lobule)		-24	-54	64	7.59	0.001	0.95
	Cerebellar Tonsil		-18	-52	-48	6.08	0.041	0.23
	Paracentral Lobule (Frontal lobe)		-16	34	50	4.49	0	0.33
	Inferior Parietal Lobule		-48	-36	24	4.32	0.022	0.43
	Cerebellar Tonsil (Cerebellum Posterior Lobe)	Right	16	-54	-48	5.68	0.015	0.23
Saccades	Middle Frontal Gyrus	Left	-36	-4	50	8.11	0.001	0.57
	Postcentral Gyrus	Right	40	-30	42	7.55	0.001	0.43
	Medial Frontal Gyrus		8	-28	68	6.02	0.034	0.26
Anti-Saccades	BA7 (Precuneus)	Left	-20	-62	54	8.34	0	0.87
	Inferior Parietal Lobule	Right	38	-34	40	9.76	0	0.58
	BA6 (Middle Frontal Gyrus)		28	-2	48	8.09	0	0.54
Optokinetic Nystagmus	Middle Temporal Gyrus	Left	-44	-70	8	10.85	0	0.99
	Precuneus		-24	-50	54	7.85	0.001	0.65
	Cerebellar Tonsil		-14	-54	-48	6.67	0.011	0.32
	BA24 (Anterior cingulate cortex)		-14	-18	42	6.41	0.19	0.25
	Postcentral Gyrus		-52	-22	38	3.83	0.024	0.40
	BA 6 (Middle Frontal Gyrus)	Right	28	-2	50	9.14	0	0.67

### Behavioural results

#### VR eye-tracking tasks

The results acquired using the ® with the VR-goggles eye-tracking system are presented in Table 3. This table includes mean value acquired on each metric with standard deviations as well as range of the values collected on healthy controls in this study in the column 3, and the norms for oculomotor tasks established by the software manufacturer (NeuroFlex). These norms were calculated using a sample size > = 717 healthy adults, ages 18–55. These norms continue to be updated by the software company as more data are acquired.

**Table 3:** Results on oculomotor tasks using VR system in the group of healthy controls.

Task	Mean (±SD)	Range (obtained in this study)	Norms (obtained by NeuroFlex©)
Smooth Pursuit (Head Free)			
Mean Vergence (degrees)	-0.10 (±1.05)	-2.2–2.0	-7.5–7.4
Vergence SD (degrees)	1.46 (±2.10)	-2.74–5.66	0–7.5
Mean Error (degrees)	2.54 (±0.76)	1.02–4.06	0–8.8
Number of Saccades (saccades)	14.83 (±7.61)	<30.05	<31
Head Contribution (%)	88.51 (±17.10)	54.31–122.71	>60
Smooth Pursuit (Head Fixed)			
Mean Vergence (degrees)	0.56 (±0.80)	-1.04–2.16	-3–3.8
Vergence SD (degrees)	1.76 (±1.10)	-0.44–3.96	0–7
Mean Error (degrees)	2.17 (±0.60)	0.97–3.37	0–7.4
Number of Saccades (saccades)	15.16 (±10.75)	<36.66	<31
Vor (Horizontal)			
Mean Vergence (degrees)	0.30 (±1.30)	-2.3–2.9	-7.4–8.1
Vergence SD (degrees)	1.93 (±1.52)	-1.11–4.97	0–7.2
Gain Left (%)	92.81 (±7.45)	77.91–107.71	67–113
Gain Right (%)	93.05 (±7.70)	77.65–108.45	66–112
VOR (Vertical)			
Mean Vergence (deg)	0.09 (±0.72)	-1.35–1.53	-6.8–7.1
Vergence SD (degrees)	0.98 (±0.43)	0.12–1.84	0–5.2
Gain Up (%)	90.94 (±6.69)	77.56–104.32	66–110
Gain Down (%)	88.95 (±8.23)	72.49–105.41	61–110
Saccades			
Mean Vergence (deg)	0.03 (±0.64)	-1.25–1.31	-5.4–5.5
Vergence SD (deg)	1.26 (±0.57)	0.12–2.4	0–6.6
Acquisition Error (deg)	2.15 (±0.68)	0.79–3.51	0–9.4
Mean Latency (ms)	244.90 (±26.98)	190.94–298.86	0–308
Anti-Saccades			
Mean Vergence (degrees)	0.42 (±0.82)	-1.22–2.06	-6.4–7.1
Vergence SD (degrees)	1.66 (±1.36)	-1.06–4.38	0–7.7
Acquisition Error (degrees)	5.71 (±2.85)	0.01–11.41	0–12
Mean Latency (ms)	464.13 (±103.32)	257.49–670.77	0–664
Directional Accuracy (%)	70.16 (±23.84)	22.48–117.84	>30
OKN			
Mean Vergence (degrees)	-0.13 (±1.84)	-3.81–3.55	-8.3–6.9
Vergence SD (degrees)	2.15 (±1.04)	0.07–4.23	0–8.6
Gain Left (%)	77.05 (±10.50)	56.05–98.05	49–110
Gain Right (%)	77.38 (±13.49)	50.4–104.36	46–112
Gain Up (%)	68.02 (±12.58)	42.86–93.18	39–102
Gain Down (%)	69.23 (±12.75)	43.73–94.73	36–101
SPN			
Mean Tremor Frequency (saccades/second)	0.25 (±0.18)	<0.61	<0.9
Average Drift (degrees/second)	0 (±0)	0	-
Mean Tremor Velocity (degrees/second)	56.10 (±31.78)	<119.66	-

### Regression analysis BOLD signal vs. oculomotor metrics

The regression analysis showed a positive correlation between the metric “Gain Down” of the OKN task and three highlighted regions of interest (i.e., activation peaks): cingulate gyrus (p = 0.019), cerebellar tonsil (p = 0.03) and postcentral gyrus (p = 0.001) (Table 4). Other comparisons did not reveal any significant results.

**Table 4:** Results of regression analysis.

Region	Task	Metric	p value
BA24 (Cingulate Gyrus)			0.019
Cerebellar Tonsil	OKN	Gain Down	0.03
Postcentral Gyrus			0.001

## Discussion

In the present study, we have explored the diagnostic potential of a set of oculomotor tasks using VR-goggles, an eye-tracking system, and fMRI. The data acquired from 31 healthy controls aimed to evaluate the range of normal performances on the oculomotor tasks and to determine regions of brain activation related to these tasks for further investigation with concussed subjects. During the oculomotor tasks, the following peak regions of activation in the healthy controls were identified: (1) **Smooth pursuit**–cuneus, superior parietal lobule, paracentral lobule, inferior parietal lobule and cerebellar tonsil, (2) **Saccades**–middle frontal gyrus, postcentral gyrus and medial frontal gyrus, (3) **Anti-saccades**—precuneus, inferior parietal lobule, and middle frontal gyrus, and, finally, (4) **OKN**—middle temporal gyrus, anterior cingulate cortex, postcentral gyrus, middle frontal gyrus, and cerebellar tonsil. These results were all associated with normal performance on the tasks.

### Areas involved in oculomotor functions

The brain regions identified in this study are in line with many of the well-known studies measuring smooth pursuit, OKN, voluntary saccades and anti-saccades [8,17–20]. The coordinates of the activation peaks obtained in this study were compared to those of previous studies (Table 5) to identify if anatomical coordinates indicated in this study were corresponding with functional areas, especially related to implementation of oculomotor movements. Thus, according to previous findings, following functional areas were involved in tasks implementation: 1) **Smooth Pursuit**: there was increased activation in the left PEF [8]; 2) **Saccades**: increased activation in the left superior FEF [21] and right SEF [18]; 3) **Anti-Saccades**: activations were seen in the PEF bilaterally [8,21] and the right superior FEF [8,20,21]; 4) **OKN**: increased activation in the left MT [22], PEF [8], CEF [19] and right Superior FEF [8,20,21].

**Table 5:** Eye fields corresponding to activation peaks in the current study.

Task	Peaks	Hemisphere	Eye Field	x	y	z
Smooth Pursuit	Cuneus	Left	V1	-10	-98	14
	BA7 (Superior parietal lobule)		PEF (Berman et al., 1999 [8])	-24	-54	64
Saccades	Middle Frontal Gyrus	Left	Superior FEF (Luna et al., 1998 [21])	-36	-4	50
	Medial Frontal Gyrus	Right	SEF (Sweeney et al., 1996 [18])	8	-28	68
Anti-Saccades	BA7 (Precuneus)	Left	PEF (Berman et al., 1999 [8])	-20	-62	54
	Inferior Parietal Lobule	Right	PEF (Luna et al., 1998 [21])	38	-34	40
	BA6 (Middle Frontal Gyrus)		Superior FEF (Luna et al., 1998 [21]; Berman et al., 1999 [8]; Kimmig et al., 2001) [20]	28	-2	48
Optokinetic Nystagmus	Middle Temporal Gyrus	Left	MT (Kolster, Peeter & Orban, 2010 [22])	-44	-70	8
	Precuneus		PEF (Berman et al., 1999 [8])	-24	-50	54
	BA24 (Anterior cingulate cortex)		CEF (Koval et al., 2014 [19])	-14	-18	42
	BA 6 (Middle Frontal Gyrus)	Right	Superior FEF (Luna et al., 1998 [21]; Berman et al., 1999 [8]; Kimmig et al., 2001) [20]	28	-2	50

Furthermore, other areas less known for their involvement in oculomotor movement were identified as activation peaks. For instance, an activation peak in tonsil complex of the cerebellum was observed during Smooth Pursuit, as well as during the OKN task, where Smooth Pursuit eye movements play a significant role. Previous studies have shown activation in the tonsil complex of the cerebellum, attributing it to primarily high-frequency, transient vestibular responses, and for smooth pursuit maintenance and steady gaze holding [23]. In addition, in monkeys, impairment of the tonsil complex homologue (the flocculus/paraflocculus) led to impaired smooth pursuit, and incomplete suppression of an induced but unwanted vestibular nystagmus [24].

Additionally, activation peaks were found in such areas as cuneus (in Smooth Pursuit) and inferior parietal lobule (Anti-Saccades). According to a review by Kobayashi, visual information is processed through the dorsal visual pathway, reaching the inferior parietal lobule, the intraparietal sulcus and the precuneus [25]. Pierrot-Deseilligny et al. showed that the inferior parietal lobule is involved in the visuospatial integration used for calculating saccade amplitude [26]. In addition, the inferior parietal and ventral occipital cortices are involved in trans saccadic processing of visual object orientation [27] and the cuneus holds a specific role in spatial frequency processing which contributes significantly to pursuit eye movements [28].

Moreover, during the OKN task, an activation peak was identified in BA24 (anterior cingulate cortex), which is attributed to Cingulate Eye Field (CEF). CEF is less discussed in the literature than traditional eye fields such as the FEF, SEF and PEF. It has been suggested that CEF controls early activation of the frontal ocular motor and premotor areas in the brainstem [17,29]. Earlier fMRI studies provided evidence for an involvement of the anterior cingulate cortex in OKN [8,30]. In addition, Dieterich et al. have described activations in BA 24 in 10 subjects during OKN [30], a result in keeping with our findings.

Activation in the postcentral gyrus was detected during the Saccades and OKN tasks and this region has been involved in saccadic movements [31,32]. In the present study, we saw activation of the postcentral gyrus in both saccades and OKN, which can be explained by the fact that saccadic eye movements are one of the components of OKN.

Finally, the observed increase in BOLD signal in the paracentral lobule is consistent with previous findings by Gurler [33] and Agtzidis et al. [34], which detected activation in this area during smooth pursuit and saccadic eye movements.

Overall, these findings contribute to our understanding of the neural mechanisms underlying different types of eye movements and suggest that these movements involve complex interactions between multiple brain regions.

### 4.2. Areas affected in concussion and future directions

The underlying neurophysiology of oculomotor functions can be disrupted following mTBI [5]. Previous studies showed difficulties in smooth pursuit, saccades, and anti-saccades in concussed patients, as well as alterations in BOLD signals in related functional brain areas (e.g., cerebellum, frontal lobes, primary and secondary visual cortex, and visual area V5/MT) [35]. Elevated activation during performance of saccadic movements in concussed subjects compared to controls has also been seen, which implies that compensatory mechanisms maintain functional performance when minor deficits in the networks are present [35].

The activation peaks identified in the current study are for the majority overlapping with many of the affected areas in concussion (such as middle temporal gyrus, cingulate cortex, precuneus, middle frontal gyrus, inferior parietal lobule, visual cortex, cerebellum, postcentral gyrus, medial frontal gyrus, superior parietal lobule, etc.) according to previous fMRI studies [36–46]. Given that these areas are involved in the implementation of the oculomotor tasks described in this study, we are confident that these tasks can be a sensitive tool in the evaluation of visual functional deficits in the diagnosis of concussion.

Based on ROI analysis and the areas of significant alterations of BOLD signal in the current study, these ROIs can be highlighted for future studies investigating concussed patients. According to the results of this pilot study and based on the findings from previous studies (8, 17, 19–21, 35–46), we identify ROIs for each task described in Table 6.

**Table 6:** Potential ROIs.

Task	Left	x	y	z	Right	x	y	z
**Smooth Pursuit**	Superior parietal lobule (BA7)	-24	-54	64	Cerebellar Tonsil	16	-54	-48
	Cerebellar Tonsil	-18	-52	-48				
	Paracentral Lobule	-16	34	50				
	Inferior Parietal Lobule	-48	-36	24				
**Saccades**	Middle Frontal Gyrus	-36	-4	50	Postcentral Gyrus	40	-30	42
					Medial Frontal Gyrus	8	-28	68
**Anti-Saccades**	Precuneus (BA7)	-20	-62	54	Inferior Parietal Lobule	38	-34	40
					Middle Frontal Gyrus (BA6)	28	-2	48
**Optokinetic Nystagmus**	Middle Temporal Gyrus	-44	-70	8	Middle Frontal Gyrus (BA6)	28	-2	50
	Precuneus	-24	-50	54				
	Cerebellar Tonsil	-14	-54	-48				
	Anterior cingulate cortex (BA24)	-14	-18	42				
	Postcentral Gyrus	-52	-22	38				

Our study has the potential to identify novel brain areas involved in eye-movements related to Optokinetic Nystagmus in concussed individuals. To our knowledge, there are no studies using this task with fMRI in such a patient group. As OKN requires multiple components for its execution (Smooth Pursuit eye movements, saccadic eye movements, VOR), it also involves the highest number of brain areas (middle temporal gyrus, precuneus, cerebellar tonsil, anterior cingulate cortex, postcentral gyrus, and middle frontal gyrus) in comparison to other tasks in this study (i.e., smooth pursuit, saccades, or anti-saccades). It also was the only task which showed significant results in regression analysis, showing potential involvement of several brain regions (left cingulate gyrus, postcentral gyrus and cerebellar tonsil) in the process of eye movements in the act of gaining down. Of note, in previous studies, it was demonstrated that cerebellar tonsil is crucial to VOR and pursuit gain [47].

Our ROIs coordinates are in keeping with those of previous studies on brain activation in oculomotor tasks using fMRI. The role of these areas in oculomotor function is also highlighted in multiple studies on concussion throughout the past decade. At this point, we predict that concussed individuals will have alterations in BOLD activation when completing oculomotor tasks and that their performances will be altered when executing the tasks through use of the VR goggles. These results suggest that the involvement of brain areas susceptible to mTBI in implementing these tasks, taken together with commonly reported oculomotor difficulties post-concussion, may lead to finding objective biomarkers using Neuroflex® eye-tracking tasks.

## Supporting information

S1 File (XLSX)
